# Regulatory impairment in untreated Parkinson’s disease is not restricted to Tregs: other regulatory populations are also involved

**DOI:** 10.1186/s12974-019-1606-1

**Published:** 2019-11-11

**Authors:** Diana D. Álvarez-Luquín, Asiel Arce-Sillas, Jaquelín Leyva-Hernández, Edgar Sevilla-Reyes, Marie Catherine Boll, Esteban Montes-Moratilla, Viridiana Vivas-Almazán, Citzielli Pérez-Correa, Ulises Rodríguez-Ortiz, Raquel Espinoza-Cárdenas, Gladis Fragoso, Edda Sciutto, Laura Adalid-Peralta

**Affiliations:** 1 Unidad Periférica para el Estudio de la Neuroinflamación en Patologías Neurológicas del Instituto de Investigaciones Biomédicas en el Instituto Nacional de Neurología y Neurocirugía, Insurgentes Sur 3877 La Fama, 14269 Ciudad de México, México; 2 Clinica de Investigación en Enfermedades Infecciosas (CIENI), Instituto Nacional de Enfermedades Respiratorias, Calz. de Tlalpan 4502, Seccion XVI, 14080 Ciudad de México, México; 3 Instituto Nacional de Neurología y Neurocirugía, Insurgentes Sur 3877 La Fama, 14269 Ciudad de México, México; 4 Departamento de Inmunología, Instituto de Investigaciones Biomédicas, Universidad Nacional Autónoma de México, Ciudad Universitaria, Circuito Escolar, Ciudad de México, México

**Keywords:** Parkinson’s disease, Untreated patients, Tregs, Bregs, CD8regs, Peripheral immune response

## Abstract

**Background:**

Parkinson’s disease (PD) is the second most common neurodegenerative disease in the world. Various studies have suggested that the immune response plays a key role in this pathology. While a predominantly pro-inflammatory peripheral immune response has been reported in treated and untreated PD patients, the study of the role of the regulatory immune response has been restricted to regulatory T cells. Other immune suppressive populations have been described recently, but their role in PD is still unknown. This study was designed to analyze the pro and anti-inflammatory immune response in untreated PD patients, with emphasis on the regulatory response.

**Methods:**

Thirty-two PD untreated patients and 20 healthy individuals were included in this study. Peripheral regulatory cells (CD4+Tregs, Bregs, CD8+Tregs, and tolerogenic dendritic cells), pro-inflammatory cells (Th1, Th2, and Th17 cells; active dendritic cells), and classical, intermediate, and non-classical monocytes were characterized by flow cytometry. Plasmatic levels of TNF-α, IFN-γ, IL-6, GM-CSF, IL-12p70, IL-4, IL-13, IL-17α, IL-1β, IL-10, TGF-β, and IL-35 were determined by ELISA.

**Results:**

Decreased levels of suppressor Tregs, active Tregs, Tr1 cells, IL-10-producer CD8regs, and tolerogenic PD-L1+ dendritic cells were observed. With respect to the pro-inflammatory response, a decrease in IL-17-α and an increase in IL-13 levels were observed.

**Conclusion:**

A decrease in the levels of regulatory cell subpopulations in untreated PD patients is reported for the first time in this work. These results suggest that PD patients may exhibit a deficient suppression of the pro-inflammatory response, which could contribute to the pathophysiology of the disease.

## Introduction

Parkinson’s disease (PD) is a neurodegenerative disorder characterized by bradykinesia, rigidity, and rest tremor. In the most severe cases, it results in a total inability to move [1]. PD is the second most common neurodegenerative syndrome after Alzheimer’s disease, affecting 1–2% of the population over 60 years old. A recent meta-analysis on 47 global epidemiological studies conducted between 1985 and 2010 showed that the prevalence of PD increases with age, with a rate of 41 per 100,000 inhabitants in the 40–49-year-old group; 107 in the 50–59-year-old group; 173 in the 55–64-year-old group; 428 in the 60–69-year-old group; 425 in the 65–74-year-old group; 1087 in the 70–79-year-old group; and 1903 in subjects older than 80 years [2].

The progressive degeneration of dopaminergic neurons in PD has been linked to different mechanisms, including α-synuclein aggregation, oxidative stress, mitochondrial and proteasome ubiquitin dysfunction, apoptosis, and neuroinflammation. Recently, a connection between the central nervous system (CNS) and the peripheral immune system has been reported in mice and humans [3, 4]; a lymphatic vessel network in the dura mater collects cerebrospinal fluid (CSF) and interstitial fluid from the subarachnoid space and brain parenchyma. Dural lymphatic vessels transport fluids and cells to deep cervical lymphatic nodes. This connection stresses the need of studying the role of the peripheral immune system in neurological diseases. CNS alterations observed in PD could lead to changes in the periphery. Several studies have reported a decrease in CD3+, CD4+, CD19+, activated T cells (CD4+CD25+), CD4+ T cells, effector cells (CD31+CD4+), regulatory T cells (Tregs) (CD4+CD25^hi^ and CD3+CD4+CD25^hi^CD127^dim^), myeloid dendritic cells (DC) (CD11c+human leukocyte antigen complex+ [HLA-DR]), and T helper (Th) cells (Th2 and Th1) in the peripheral blood [5–11]. Other reports showed an increase in CD4−CD8+ cells, memory cells (CD45RO+CD95+CD4+), natural killers (NKs), monocyte precursor cells, and classical monocytes, as well as in Th1 and Th17 cells [6–8, 10, 12, 13].

Other studies have reported peripheral alterations in cytokine levels, namely increased levels of the tumor necrosis factor alpha (TNF-α), interleukin- (IL-) 1β, IL-2, and IL-10 [14–17] and decreased levels of IL-4, IL-6, IL-10, TNF, interferon gamma (IFN-γ), and IL-17α [5, 6, 14]. Conflicting results on the levels of some molecules and immune populations have been reported, possibly due to differences in treatment and in the clinical status of the subjects included [5, 6, 8, 10, 18, 19]. Few studies have described the immune response in untreated patients [18, 20–22].

Altogether, these findings suggest that the peripheral pro-inflammatory immune response has an important role in the progression of PD. In fact, both the levels of cytokines and of immune cells are related to symptom worsening, as reflected in clinical scales like the Hoehn & Yahr (H&Y) scale and the Unified Parkinson’s Disease Rating Scale (UPDRS) [10, 23]. This persistent inflammation in PD patients may be suppressed by regulatory cell populations. However, there are few studies about the immunomodulatory response in PD patients.

With respect to the suppression of the immune response, only a regulatory response mediated by T-CD4 regulatory cells has been reported in PD. While some reports have described a decline in Tregs populations, other studies reported no changes. Cells with the phenotype CD4+CD25+, CD4+CD25^hi^, CD4+CD25+FOXP3+, CD4+CD25+CD127−, and CD3+CD4+CD25^hi^CD127^dim^ have been labeled as Tregs [5–8, 10]. In addition, experimental data from murine PD models support the role of Tregs in neuroprotection [24].

Other regulatory populations, like regulatory B cells (Bregs), CD8 regulatory T cells (CD8regs), and plasmatic cells, can also produce IL-10 and have suppressive actions [25–27]. In addition, tolerogenic DCs induce, convert, or expand Tregs [28]. However, no study has described yet the role of these regulatory populations in PD.

Monocytes can be categorized as classical, intermediate, and non-classical according to their expression of CD14 and CD16, exhibiting pro-inflammatory properties in a higher or lesser degree and even anti-inflammatory traits [29]. No study has analyzed yet the role of these monocyte populations in PD.

Thus, considering the possible relevance of inflammation in the pathogenesis of PD, the anti- and pro-inflammatory immune response in untreated PD patients was analyzed in this work, with emphasis on the regulatory response.

## Material and methods

Thirty-two patients with no previous dopaminergic treatment and 22 healthy subjects (controls) were included in this study. All patients attended the Instituto Nacional de Neurología y Neurocirugía (INNN) and agreed to participate in this study by signing an informed consent letter. The protocol was approved by the INNN Ethics Committee (permit No. 95/14) and was conducted in accordance with the Helsinki Declaration. All diagnoses were performed by an expert neurologist at the INNN, following the United Kingdom Parkinson’s Disease Society Brain Bank (UK PDSBB) diagnosis criteria. The UPDRS and H&Y scales, as well as the Schwab & England and Beck depression questionnaires, were applied to comprehensively evaluate the patients’ clinical status and state of mind.

### Samples

Twenty milliliters of peripheral blood were collected from each patient and control subject in tubes containing acid-citrate-dextrose (ACD) (Vacutainer ACD, BD Franklin Lakes, NJ, USA). Blood samples were centrifuged to separate the plasma, and the cells were resuspended in sterile phosphate buffer solution (PBS) 1×. The samples were diluted 1:1 with sterile PBS 1×. Peripheral blood mononuclear cells (PBMCs) were separated by density gradient using Ficoll-Hypaque (Sigma Aldrich, Little Chalfont, UK). The samples were centrifuged for 30 min at 1800 rpm, without brake. Subsequently, the PBMCs were separated and washed with PBS 1× twice, centrifuging for 10 min at 1600 rpm. Finally, the cells were resuspended in 1 mL of 1× PBS and counted in a Neubauer chamber using Trypan Blue stain. Only samples with at least 95% of cellular viability were used to characterize cellular phenotypes by flow cytometry.

### Cell population labeling for flow cytometry analysis

For each phenotype and isotype, 10^6^ PBMCs in a total volume of 50 μL were stained and analyzed. For intracellular labeling, PBMCs were incubated with brefeldin (10 μg/mL) for 4 h at 37 °C and 5% CO_2_ before labeling. Cytokine production was assayed in unstimulated PBMCs, since it has been reported that cytokine induction heavily depends on the stimulus used [30]; additionally, the analysis of unstimulated PBMCs has been reported previously [31–33]. Thus, only spontaneous cytokine production was considered in this study. Thereafter, antibodies for extracellular labeling were added and incubated for 30 min at 4 °C. PBMCs were washed with PBS supplemented with bovine serum albumin 5% and fetal bovine serum 1%. Then, PBMCs were permeabilized by adding 300 μL of fixation and permeabilization buffers (eBioscience, Waltham, MA, USA). PBMCs were then incubated at 4 °C for 2 h, washed with permeabilization buffer (eBioscience), blocked with 20 μL of 10% rat serum solution, and incubated again for 1 h at 4 °C. Intracellular antibodies were added with no previous wash, and PBMCs were incubated for 30 min at 4 °C. Finally, PBMCs were washed with permeabilization buffer and fixed with 200 μL of 2% paraformaldehyde.

Cell phenotype antibodies. CD4 regulatory T cells: CD127 fluorescein isothiocyanate (FITC)^a^ (isotype mouse IgG1k FITC), FOXP3 phycoerythrin (PE)^a^* (isotype rat IgG2ak PE), IL-10 PE^a^* (isotype rat IgG2ak PE), CD45RO Peridinin-chlorophyll protein complex-cyanine 5.5 (PerCP Cy5.5)^c^ (isotype mouse IgG2ak PerCP Cy5.5), TGF-β PerCP Cy5.5^d^* (isotype mouse IgG1k PerCP Cy5.5), CD25 allophycocyanin (APC)^b^ (isotype mouse IgG1k APC), and CD4 allophycocyanin-cyanine 7 (APC Cy7)^b^ (isotype mouse IgG1k APC Cy7). The markers analyzed for each Tregs phenotype are shown in Additional file 1: Table S1. As shown, different Tregs cell phenotypes already described in the literature were analyzed, because different subsets of Tregs have been reported to suppress the immune response through different mechanisms [34–37]. CD8 regulatory T cells: CD56 FITC^a^ (isotype mouse IgG1k FITC), CCR7 FITC^a^ (isotype rat IgG2ak FITC), FOXP3 PE^a^* (isotype rat IgG2ak PE), IL-10 PE^a^* (isotype rat IgG2ak PE), CD161 PerCP^a^ (isotype mouse IgG1 PerCP Cy5.5), CD45RO PerCP^c^ (isotype mouse IgG2ak PerCP), CD8 APC^a^ (isotype mouse IgG1k APC), and CD28 allophycocyanin-Hilite®7 (APC H7)^b^ (isotype mouse IgG1k APC H7). With respect to CD8regs, different phenotypes were analyzed as well [25–27] Additional file 1: Table S1. B regulatory and plasma cells: CD138 FITC^a^ (isotype mouse IgG1k FITC), CD5 FITC^a^ (isotype mouse IgG2ak FITC), FOXP3 PE^a^* (isotype rat IgG2ak PE), IL-10 PE^a^* (isotype rat IgG2ak PE), CD24 PerCP^b^ (isotype mouse IgG2ak PerCP Cy5.5), CD1d, Peridinin chlorophyll proteins complex-eFluor 710 (PerCP eFluor 710)^b^ (isotype mouse PerCP eFluor 710), CD38 APC^a^ (isotype mouse IgG1k APC), IL-10 APC^b^* (isotype rat IgG2a APC), and CD19 APC Cy7^b^ (isotype mouse IgG1k APC Cy7). Different phenotypes were analyzed for Bregs and plasmatic cells [38, 39] (Additional file 1: Table S1). Monocytes (classical, non-classical, and intermediate): CD16 FITC^a^ (isotype mouse IgG1k FITC), IL-12 PE^b^* (isotype mouse IgG1 PE), IL-10 PE^a^* (isotype rat IgG2ak PE), CD14 PerCP^a^ (isotype mouse IgG1k PerCP Cy5.5), CD163 APC^a^ (isotype mouse IgG1k APC), and HLA-DR APC Cy7^b^ (isotype mouse IgG2ak APC Cy7). With respect to monocytes, the phenotypes proposed by Ziegler-Heitbrock and Wong [29, 40, 41] were analyzed (Additional file 1: Table S1). Dendritic cells: programmed death-ligand 1 (PD-L1) FITC^b^ (isotype mouse IgG1k FITC), CD205 FITC^a^ (isotype mouse IgG2bk FITC), signaling lymphocytic activation molecule (SLAM) PE^a^ (isotype mouse IgG1k PE), CD40 PE^b^ (isotype mouse IgG1k PE), CD11c PerCP eFluor 710^a^ (isotype mouse IgG1k PerCP eFluor 710), immunoglobulin-like transcript 3 (ILT3) APC^a^ (isotype mouse IgG1 APC), CD86 APC^b^ (isotype mouse IgG1 APC), HLA-DR APC Cy7^b^ (isotype mouse IgG2ak APC Cy7), and CD80 APC H7^b^ (isotype mouse IgG1k APC H7). For DCs, different phenotypes reported in the literature were analyzed [34] (Additional file 1: Table S1). Th1 cells: IFN-γ FITC^a^* (isotype mouse IgG1k FITC), Tbet PE^a^* (isotype mouse IgG1k PE), TNF-α PerCP Cy5.5^a^* (isotype mouse IgG1k PerCP Cy5.5), and CD4 APC Cy7^b^ (isotype mouse IgG1k APC Cy7). Th1 phenotypes are described in Additional file 1: Table S1, [42–44]. Th2 cells: IL-13 FITC^a^* (isotype mouse IgG1k FITC), IL-4 PE^a^* (isotype mouse IgG1k PE), GATA-3 PerCP^a^* (isotype rat IgG2bk PerCP eFluor 710), and CD4 APC Cy7^b^ (isotype mouse IgG1k APC Cy7). Th2 phenotypes are shown in Additional file 1: Table S1 [42–44]. Th17 cells: IL-17α FITC^a^* (isotype mouse IgG1k FITC), ROR-γ (t isoform) PE^a^* (isotype rat IgG2a PE), and CD4 APC Cy7^b^ (isotype mouse IgG1k APC Cy7). The Th17 phenotype is shown in Additional file 1: Table S1 [42, 43, 45, 46]. Isotype controls were used to discriminate positive from negative cells. Finally, the antibody labeling and the respective isotype controls for all antibodies used in this work are shown in Additional file 2: Figure S1. The antibodies used in this study were purchased from eBioscience (Waltham, MA, USA) (^a^), BD (Franklin Lakes, NJ, USA) (^b^), Invitrogen (Waltham, MA, USA) (^c^), and Biolegend (San Diego, CA, USA) (^d^); *intracellular antibodies. Labeled cells were read in an Attune Acoustic Focusing Cytometer (Applied Biosystems, Waltham, MA, USA) and analyzed with the Attune Cytometric Software v.1.2.5. The strategy used in the flow cytometry analysis is shown in Additional file 3: Figure S2.

### Cytokines

Cytokine levels were determined by enzyme-linked immunosorbent assay (ELISA) in plasma samples from patients and controls. An Elabscience kit (Wuhan, China) was used for IL-35 determination, following the manufacturer’s instructions. Invitrogen kits were used to determine the levels of the granulocyte-macrophage colony-stimulating factor (GM-CSF), IFN-γ, IL-1b, IL-4, IL-6, IL-10, IL-12p70, IL-13, IL-17α, the transforming growth factor beta (TGF-β), and TNF-α, following the manufacturer’s protocols. TGF-β was measured using the TGF-β1 ELISA Ready-Set-Go, which includes an acid treatment to detect both the mature cytokine and the TGF-β1 latency-associated peptide (LAP). The detection limits were 2 pg/mL for IL-1β, IL-4, IL-6, and IL-10; 4 pg/mL for IL-13, IFN-γ, TNF-α, IL-12p70, and IL-17α; 6 pg/mL for GM-CSF; 8 pg/mL for TGF-β; and 9.38 pg/mL for IL-35.

### Statistical analysis

The Mann-Whitney *U* test was used to compare the results in patients and controls. All correlations were analyzed with the Spearman test. A multiple test correction following the Holman-Bonferroni method was performed for all populations. Corrected values are reported only when significance was lost.

## Results

### Clinical characteristics of patients and controls

None of the patients included in this study had received treatment for PD before enrollment. The clinical and physiopathological characteristics of patients and controls are described in Table 1. The patients had an H&Y mean score of 2.17, indicating a mild motor stage of the disease. The mean UPDRS score in patients was 51.59. The mean duration of symptoms was 2.90 ± 3.14 years. According to the Schwab-England scale, the patients were completely independent to do most chores. On the other hand, the UPDRS mean score for control subjects was 2.77, as expected in any healthy person. Although the patients had a significantly higher score in Beck’s scale (5.54 ± 6.24 for controls and 11.50 ± 8.00 for PD patients, *P* = 0.003), neither patients nor controls showed depression. In fact, clinical depression is defined as a score higher than 13 in that scale. Data on hemogram, blood chemistry, and hormonal profile for patients and controls are shown in Additional file 4: Table S2, Additional file 5: Table S3 and Additional file 6: Table S4. The clinical inflammatory status in patients and controls, as assessed by the levels of C-reactive protein (CRP) and erythrocyte sedimentation rate (ESR) (0.52 ± 0.88 and 0.19 ± 0.24 for CRP and 21.62 ± 16.26 and 16.87 ± 14.30 for ESR, respectively), showed no significant differences between both groups.

**Table 1:** Clinical features of patients and controls

	Control	PD	*P* value
Socio-demographic and clinical characteristics			
Age at inclusion^¢^	55.59 ± 10.22	60.81 ± 10.23	0.064
Male:female ratio^	54:46	56:44	–
Body mass index (BMI)^¢^	26.85 ± 3.89	27.52 ± 4.54	0.549
Symptom duration (years)^¢^	NA	2.90 ± 3.14	–
Tremoric:akinetic ratio^	NA	59.4:53.1	–
Clinical scales^¢^			
Hoehn & Yahr	NA	2.17 ± 0.88	–
UPDRS I	0.72 ± 0.98	2.50 ± 1.60	< 0.0001***
UPDRS II	0.32 ± 0.89	13.28 ± 6.56	< 0.0001***
UPDRS III	1.72 ± 3.71	35.68 ± 18.29	< 0.0001***
UPDRS IV	0 ± 0	0 ± 0	> 0.999
Total UPDRS	2.77 ± 4.05	51.59 ± 25.13	< 0.0001***
Schwab-England scale	99.54 ± 2.13	76.59 ± 22.55	< 0.0001***
Beck’s depression test	5.54 ± 6.24	11.50 ± 8.00	0.003**
Non-motor symptoms^^^			
Sialorrhea	NA	25	–
Sleep disturbance	NA	50	–
Hyposmia	NA	25	–
Depression	NA	50	–
Falls	NA	25	–
Comorbidities^^^			
Diabetes mellitus type 2	13.6	12.5	0.999
Hypertension	9.1	37.5	0.029*
Dyslipidemia	0	12.5	0.141
Depressive disorder	3.1	6.25	0.557

^¢^Values are expressed as mean ± SD

^^^Values are expressed as percentage of subjects

*P < 0.05 is considered as significant

** *P* < 0.005 is considered as significant

***P < 0.0005 is considered as significant

### The levels of regulatory cells are decreased in untreated PD patients

**Fig. 1:**
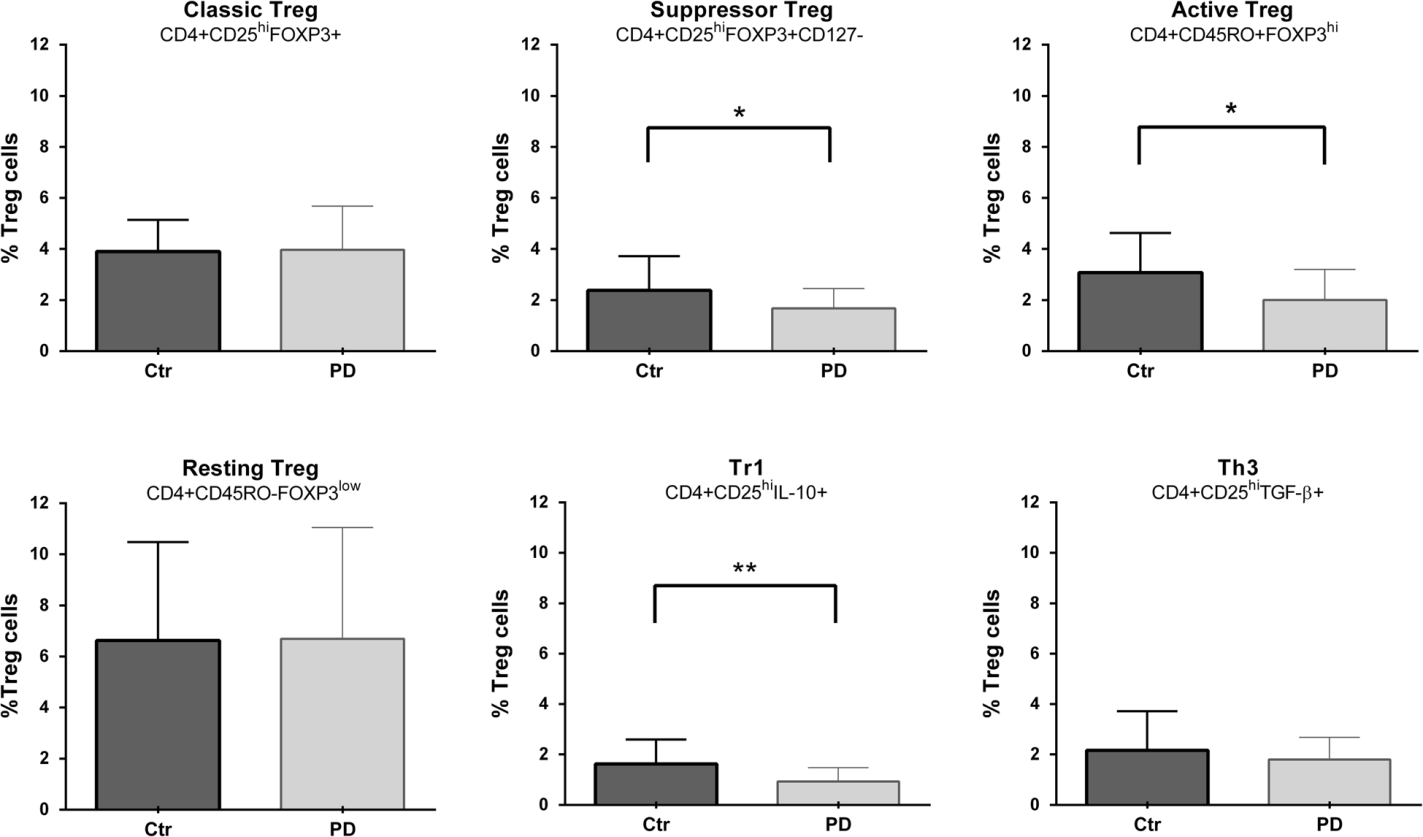
Decreased levels of active Tregs, suppressor Tregs, and Tr1 cells in PD patients. Differences between controls (Ctr) and PD patients (PD) are shown. The percentage corresponding to each cell phenotype is shown on the *y*-axis. *P <  0.05 is considered as significant, **P <  0.005 is considered as significant

To characterize the anti-inflammatory response, several populations of regulatory cells, including CD4 Tregs, Bregs, CD8regs, and tolerogenic DCs, were analyzed. PD patients exhibited a significantly lower percentage of total CD4 cells than control subjects (13.76 ± 7.05 and 17.27 ± 7.96, respectively, *P* = 0.0002, as determined from size dot plots; 33.58 ± 9.73 and 40.59 ± 9.50, respectively, *P* = 0.0001, as determined from size/dispersion dot plots). The regulatory CD4 T cell subpopulations are shown in Fig. 1. As shown, the percentage of suppressive Tregs, activated Tregs, and type-1 regulatory T cells (Tr1) was lower in patients than in controls. Interestingly, a positive correlation was found between suppressive Tregs and non-Tregs (*r* = 0.516, *P* = 0.003) and between suppressive Tregs and classical Tregs (*r* = 0.550, *P* = 0.001). With respect to CD8 regulatory T cells, the patients showed significantly lower levels of CD8 IL-10+ cells and functional CD8regs than controls (Table 2); however, when a multiple test correction was performed, the *P* value for the differences in these populations to be considered as significant is < 0.02; therefore, the decrease in the levels of functional CD8regs should be taken with caution.

**Table 2:** Levels of human CD8regs and Bregs in PD patients and healthy controls

Cell subset		Control^¢^	PD^¢^	*P* value
CD8+		16.69 ± 8.60	14.55 ± 8.05	0.209
	CD8+IL-10+	3.84 ± 2.47	2.47 ± 1.91	0.006*
	CD8regs (CD8+CD28-FOXP3)	13.21 ± 5.13	14.41 ± 5.41	0.522
	Cytolytic CD8regs (CD8+CD56+CD161-)	12.70 ± 7.03	13.08 ± 9.23	0.663
	Functional CD8regs (CD8+CD45RO+CCR7+IL-10+)	0.38 ± 0.31	0.23 ± 0.17	0.049*
CD19+		17.34 ± 6.65	17.35 ± 6.61	0.930
	CD19+IL-10+	3.22 ± 1.58	3.173 ± 1.76	0.690
	Plasmatic cells IL-10+ (CD19-CD138+IL-10+)	32.83 ± 14.04	26.13 ± 18.99	0.219
	Functional Bregs (CD19+CD38^hi^CD24^hi^IL-10+)	3.82 ± 2.16	2.64 ± 1.88	0.049*
	Bregs (CD19+CD5+CD1d+FOXP3+IL-10+)	1.49 ± 1.13	1.29 ± 0.91	0.555

^¢^Values are expressed as mean ± SD

*P < 0.05 is considered as significant

The analysis of regulatory B cell subpopulations showed that the levels of functional Bregs were significantly lower in patients than in controls (Table 2).

The multiple test correction showed that *P* <  0.02 is considered as significant for differences in this population, so this result should also be taken with caution.

Finally, tolerogenic DCs that express PD-L1 were significantly lower in patients than in controls. The other markers in DCs showed no difference between patients and controls (Table 3). However, a positive correlation between the suppressive molecule PD-L1 and CD205 was found in patients (*r* = 0.443, *P* = 0.016). HLA-DR also correlated positively with PD-L1, ILT3, and CD205 (*r* = 0.538, *P* = 0.001, *r* = 0.643, *P* = 0.0001; and *r* = 0.647, *P* = 0.0001, respectively).

**Table 3:** Levels of human dendritic cells in PD patients and healthy controls

Cell subset			Controls^¢^	PD^¢^	*P*
CD11c+			54.58 ± 17.08	52.27 ± 18.22	0.633
Dendritic cells	Tolerogenic	PD-L1+	12.63 ± 6.74	10.57 ± 9.49	0.031*
		SLAM+	9.84 ± 6.24	9.65 ± 4.73	0.890
		ILT3+	37.92 ± 18.03	41.01 ± 18.46	0.588
		CD205+	29.22 ± 15.27	30.64 ± 14.61	0.707
	Active	HLA-DR+	45.58 ± 19.33	45.48 ± 19.24	0.848
		CD40+	9.11 ± 9.59	10.45 ± 12.51	0.981
		CD86+	53.11 ± 17.15	52.44 ± 22.76	0.766
		CD80+	18.75 ± 11.17	22.86 ± 10.87	0.174

^¢^Data are expressed as mean ± SD

*Values are considered as significantly different for *P* < 0.05

### Pro-inflammatory cell levels in PD patients

The levels of Th1, Th2, and Th17 cells, as well as those of HLA-DR and costimulatory molecules in DCs were measured in both patients and controls. No significant differences were found in the levels of Th1, Th2, nor Th17 cells between patients and controls (Table 4).

**Table 4:** Levels of human CD4 T helper cells in PD patients and healthy controls

CD4 cell subset		Control^¢^	PD^¢^	*P* value
CD4+		17.27 ± 7.96	13.76 ± 7.05	0.003**
Th1	CD4+Tbet+IFN-γ+	1.98 ± 2.32	1.63 ± 1.30	0.733
	CD4+Tbet+TNF−α+	3.27 ± 2.94	1.83 ± 1.62	0.165
Th2	CD4+Gata-3+IL-13+	3.49 ± 5.32	4.94 ± 7 .21	0.537
	CD4+Gata-3+IL-4+	2.43 ± 3.71	2.45 ± 2.93	0.455
Th17	CD4+Ror-γ+IL-17α+	1.47 ± 2.16	0.98 ± 0.78	0.412

^¢^Values are expressed as mean ± SD

*P < 0.05 is consider as significant

**P < 0.005 is consider as significant

CD4+ cells were determined as described in the “Material and methods” section for T helper cells

No differences were found in the markers for active DCs between patients and controls (Table 3). However, HLA-DR was found to positively correlate with CD40 and CD80 in patients (*r* = 0.514, *P* = 0.004; *r* = 0.406, *P* = 0.029, respectively); CD86 positively correlated with CD40 (*r* = 0.432, *P* = 0.019). Finally, a positive correlation was observed between CD40 and CD80 (*r* = 0.647, *P* = 0.0001).

### Monocyte levels in PD patients

No differences in the percentage of total CD14+ cells were found between controls and patients (8.97 ± 3.55 and 9.81 ± 4.92, respectively). Monocytes were characterized by the expression of CD14 and CD16 and then subclassified into non-classical, intermediate, and classical monocytes. As shown in Additional file 7: Table S5, no differences were found between patients and controls. Additionally, no differences were observed in the percentage of M1-like cells (0.02 ± 0.03 and 0.03 ± 0.04, respectively) nor M2-like cells (0.09 ± 0.11 and 0.07 ± 0.09, respectively) between controls and patients.

### Cytokine levels in PD patients

**Fig. 2:**
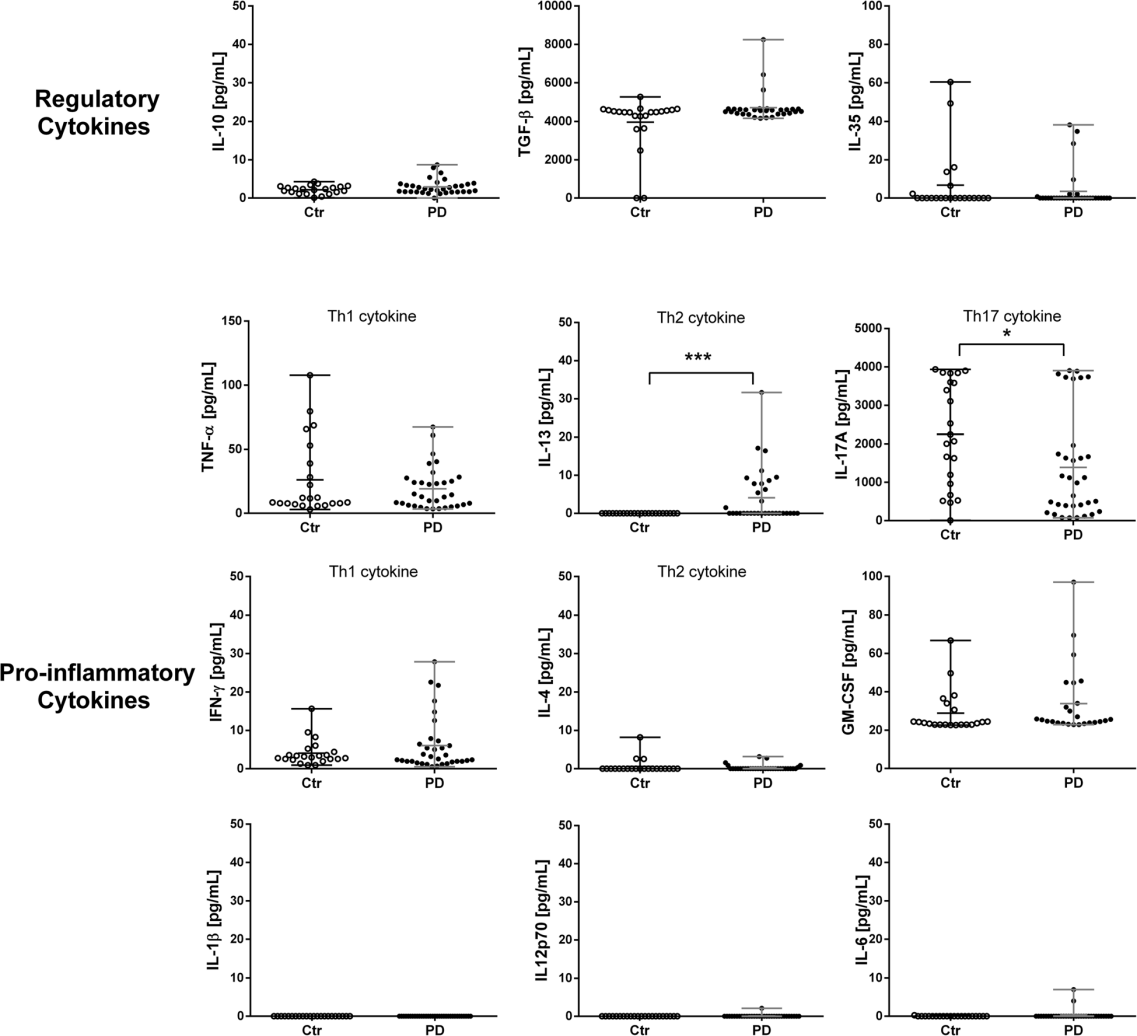
Increased plasmatic levels of IL-13 and decreased levels of IL-17A in PD patients. The plasmatic levels of regulatory (IL-10, IL-35, and TGF-β) and pro-inflammatory cytokines (IL-1β, IL-6, IL-12p70, and GM-CSF), Th1 cytokines (TNF-α and IFN-γ), Th2 cytokines (IL-4 and IL-13), and Th17 cytokines (IL-17A) are shown in controls and patients. Cytokine concentrations, expressed in pg/mL, are shown on the *y*-axis. *P < 0.05 is consider as significant, **P < 0.005 is consider as significant, and ***P < 0.0005 is consider as significant

With respect to regulatory cytokines, no significant differences were observed in the levels of TGF-β, IL-10, nor IL-35 in PD patients with respect to control subjects (Fig. 2).

Pro-inflammatory cytokines were classified according to the T helper phenotype that they are capable to polarize, or to the cytokine produced by each cell type. Thus, no significative differences were found between patients and controls for the Th1 phenotype (TNF-α and IFN-γ). For the Th2 phenotype (IL-4 and IL-13), IL-13 levels were higher in patients than in controls (*P* = 0.001). For Th17 (IL-17α), the levels of IL-17α were lower in patients than in controls (*P* = 0.014). Interestingly, the plasmatic levels of IL-17 positively correlated with the H&Y, UPDRS III, and UPDRS-total scores (*r* = 0.4090, *P* = 0.0247; *r* = 0.4162, *P* = 0.0222; and *r* = 0.3945, *P* = 0.0310, respectively).

The levels of IL-1β, IL-12p70, IL-6, and GM-CSF were not significantly different between patients and controls (Fig. 2).

## Discussion

In this study, the immunological features related to the pro- and anti-inflammatory responses were studied in peripheral blood samples from a cohort of Parkinson’s disease patients naïve to dopaminergic treatment. All enrolled patients exhibited normal laboratory test results (hemogram, blood chemistry, and hormonal profile, as shown in Additional file 4: Table S2, Additional file 5: Table S3 and Additional file 6: Table S4. No significant differences were observed in unspecific inflammatory markers (CRP and ESR) between patients and controls. These markers are acute-phase proteins produced by the liver before pro-inflammatory cytokines like TNF-α, IL-6, and IL-1β are secreted; thus, inflammation in PD patients can be considered as a chronic process.

The main alteration in the immune profile of PD patients was the composition of the population of regulatory cells. Interestingly, lower levels of suppressive Tregs (CD4+CD25^hi^FOXP3+CD127^−^), active Tregs (CD4+CD45RO+FOXP3^hi^), and Tr1 cells (CD4+CD25^hi^IL-10+) were found in patients with respect to controls. Our finding of decreased levels of suppressive Tregs is in agreement with the work by Saunders (2012), although only treated patients were enrolled in that study [7]. Therefore, the reduced levels of suppressive Tregs seem to be associated with the pathogeny of PD. With respect to active Tregs and Tr1 cells, this work is (to the best of our knowledge) the first study to report decreased levels of these cells in PD patients. A decrease in Tregs levels could have an important role in the progression of PD. The absence of Tregs has been linked to rapid cognitive deterioration, as well as to a rapid disease progression in murine models of neurodegenerative disorders like Alzheimer’s disease and amyotrophic lateral sclerosis [47–49]. These findings emphasize the relevance of decreased Tregs levels in the pathology of PD, since it is possible that Tregs are unable to control the pro-inflammatory response that is associated with a progression of the disease [7].

On the other hand, suppressor Tregs and active Tregs act by inhibiting the proliferation of effector CD4+ cells [50–52], while Tr1 cells are characterized by producing IL-10, an anti-inflammatory cytokine that suppresses several pro-inflammatory pathways involving transcription factors like the nuclear factor-kappa B (NF-κB) and those involving the mitogen-activated protein kinase (MAPK).

With respect to CD8regs lymphocytes, a decrease in the levels of IL-10-producing CD8 cells and a tendency to decrease in functional CD8regs (CD8+CD45RO+CCR7+IL-10+) was observed in PD patients. Both cell lines are mainly responsible for regulating the inflammatory response by secreting IL-10. Interestingly, the levels of IL-10-producing CD8 cells have been reported as significantly reduced in the exacerbation phase of multiple sclerosis but increased during remission, suggesting a favorable effect on patient recovery [53–55]. Thus, the role of these cells in PD progression should be further studied.

A tendency to decrease in the levels of functional Bregs (CD19+CD38^hi^CD24^hi^IL-10+) was found in PD patients. Bregs cells have been involved in the recovery of experimental autoimmune encephalomyelitis and in the induction and maintenance of Tregs [56]. Thus, the tendency to decrease in Breg levels suggests an impairment in the immunoregulatory response in PD patients. Another possible explanation for this fact is their involvement in the suppression of antibody-producing B cells, since antibodies are critically involved in PD, and a failure in their suppression could contribute to pathogeny [57].

With respect to tolerogenic DCs, a decrease in the expression of PD-L1 was observed in PD patients. This fact is remarkable, since PD-L1 is known to play an important role in the development and maintenance of Tregs and inducible Tregs (iTregs) [58, 59]. Thus, the lower expression of PD-L1 in PD patients could be related to the decreased Tregs levels found in our patients.

Altogether, the regulatory immune response in treatment naïve PD patients is characterized by decreased levels of active Tregs, suppressive Tregs, Tr1 cells, functional CD8regs, CD8 IL-10+, functional Bregs, and tolerogenic PD-L1+ DCs, which could be failing to control the pro-inflammatory response.

With regard to the pro-inflammatory response, we found decreased plasmatic levels of IL-17A and increased levels of IL-13 in patients with respect to controls. This could be associated with the type of immune response observed in patients (towards a Th2 phenotype instead of a Th17); however, this fact is not supported by any other experimental result.

Pairwise positive correlations were found between the levels of DCs and several immunomodulatory molecules (PD-L1, ILT3, and CD205); similar positive correlations were observed between DCs and molecules that activate the immune response (CD80, CD86, and CD40). This suggests that both activating and tolerogenic cell phenotypes are participating in the immune response of the patients, although no clear predominance of any specific phenotype was observed.

Considering that PD has been associated with chronic inflammatory processes, the alterations found in this work, mainly related to a deficient anti-inflammatory response, suggest that an imbalance between regulation and inflammation could be underlying the pathogeny of PD, at least in part. Indeed, the levels of several Tregs subpopulations were lower in patients than in controls, but at the same time, these Tregs showed positive correlations with other Tregs subpopulations, suggesting the existence of a feedback among various cell populations. Positive correlations of suppressive and non-Tregs and with classical Tregs could be associated to a transition of non-Tregs towards a functional Tregs phenotype to offset their lower levels, as it has been reported before [50]. The levels of IL-10-producer (Tr1) cells are lower in PD patients, as well as the levels of IL-10-producer CD8 cells. Although these findings suggest that IL-10 may not be involved in the immunosuppression observed in PD patients, further studies are required to confirm its role.

A comparative analysis between sexes, which would allow us to compare sex-related differences in the immune response, was not performed in this work. Sex has been suggested as a possible risk factor in PD, since a male/female ratio of about 2:1 has been reported in PD patients [60]. Some studies have shown that the clinical presentation of PD is slightly different in women, with a higher frequency of dyskinesia and depression. In addition, women generally are diagnosed with PD at an older age than men, possibly due to a decrease in estrogen production [60]. The lack of a comparative analysis of the immune response between sexes is a limitation of our work. Further studies on a larger cohort of male and female PD patients should be conducted to elucidate these differences.

## Conclusions

A decrease in the levels of CD4+ Tregs subpopulations, of functional Bregs, of IL-10-producing CD8+ lymphocytes, of functional CD8regs, and of tolerogenic PD-L1+ DCs in untreated PD patients is reported in this work, for the first time. The decreased levels of various phenotypes of regulatory cells suggest a deficiency in the regulatory immune response in untreated PD patients. These findings could contribute to deepen our understanding of the pathophysiology of the disease.

## Supplementary information

**Additional file 1: Table S 1.** Cell populations analyzed and their phenotypes. All the markers used to analyze all the subpopulations and the combinations are shown.

**Additional file 2: Figure S1.** Histograms of all markers used. All antibodies used to characterize the cellular populations in our study are shown. Histograms of every marker with its respect isotype were plotted. Isotypes are shown in gray, and markers are shown in blue.

**Additional file 3: Figure S2.** Gating strategies for Tregs, CD8regs, Bregs, monocytes, DCs, and T helper cells. A. CD4 regulatory T cells. B. CD8 regulatory T cells. C. B regulatory cells. D. Monocytes. E. Dendritic cells. F. T helper cells.

**Additional file 4: Table S2.** Biometric screening: Hemogram. Differences in the total cell blood count between patients and healthy controls are shown.

**Additional file 5: Table S3.** Biometric screening: Blood chemistry. Differences in blood chemistry test between patients and healthy controls are shown.

**Additional file 6: Table S4.** Biometric screening: Hormonal profile. Differences in the hormonal profile between patients and healthy controls are shown.

**Additional file 7: Table S5.** Levels of human monocytes in PD patients and healthy controls. Differences between subpopulations of monocytes between patients and healthy controls are shown.

## Data Availability

The datasets used and/or analyzed in this study are available from the corresponding author on reasonable request.

## Abbreviations

ACD: Acid-citrate-dextrose
APC Cy7: Allophycocyanin-cyanine 7
APC H7: Allophycocyanin-Hilite®7
APC: Allophycocyanin
Bregs: Regulatory B cells
CD8regs: Regulatory CD8 T cells
CIENI: Clínica de Investigación en Enfermedades Infecciosas
CNS: Central nervous system
CRP: C-reactive protein
CSF: Cerebrospinal fluid
DCs: Dendritic cells
ELISA: Enzyme-linked immunosorbent assay
ESR: Erythrocyte sedimentation rate
FITC: Fluorescein isothiocyanate
GM-CSF: Granulocyte-macrophage colony-stimulating factor
H&Y: Hoehn and Yahr
HLA: Human leukocyte antigen complex
IFN-γ: Interferon gamma
IL: Interleukin
ILT3: Immunoglobulin-like transcript 3
INNN: Instituto Nacional de Neurología y Neurocirugía
iTregs: Inducible regulatory T cells
LAP: Latency-associated peptide
MAPK: Mitogen-activated protein kinase
NF-κB: Nuclear factor-kappa B
NKs: Natural killers
PBMCs: Peripheral blood mononuclear cells
PBS: Phosphate buffer solution
PD: Parkinson’s disease
PD-L1: Programmed death-ligand 1
PE: Phycoerythrin
PerCP Cy5.5: Peridinin-chlorophyll proteins complex-cyanine 5.5
PerCP eFluor 710: Peridinin chlorophyll proteins complex-eFluor 710
SLAM: Signaling lymphocytic activation molecule
TGF-β: Transforming growth factor beta
Th: T helper
TNF-α: Tumor necrosis factor alpha
Tr1: Type-1 regulatory T cells
Tregs: Regulatory T cells
UK PDSBB: United Kingdom Parkinson’s Disease Society Brain Bank
UPDRs: Unified Parkinson’s disease rating scale
