# Supplementary material for: Regulatory impairment in untreated Parkinson’s disease is not restricted to Tregs: other regulatory populations are also involved
Source: J Neuroinflammation. 2019 Nov 11;16:212. doi: 10.1186/s12974-019-1606-1 (PMC6849192; doi:10.1186/s12974-019-1606-1)
Supplement: Supplementary file 1 — Additional file 1: Table S 1. Cell populations analyzed and their phenotypes. All the markers used to analyze all the subpopulations and the combinations are shown. [file 12974_2019_1606_MOESM1_ESM.docx]

**Supplementary Table 1. Cell populations analyzed and their phenotypes**

| **Cell** |  | **Phenotype** | **References** |
| --- | --- | --- | --- |
| **CD4 regulatory** |  | Classical Tregs: CD4+CD25+FOXP3+ | [30] |
|  |  | Active Tregs: CD4+CD45RO+FOXP3^hi^ | [30] |
|  |  | Non-Tregs: CD4+CD45RO+FOXP3^med^ | [30] |
|  |  | Resting Tregs: CD4+CD45RO-FOXP3^low^ | [30] |
|  |  | Suppressive Tregs: CD4+CD25^hi^FOXP3+CD127- | [31,33] |
|  |  | Tr1: CD4+CD25^hi^IL-10+ | [31] |
|  |  | TH3: CD4+CD25^hi^TGF-β+ | [32] |
| **CD8 regulatory** |  | Cytolytic CD8regs: CD8+CD56+CD161- | [25] |
|  |  | CD8regs: CD8+CD28-FOXP3+ | [26] |
|  |  | Functional CD8regs: CD8+CD45RO+CCR7+IL-10+ | [27] |
| **B regulatory** |  | IL-10-producing plasma cells: CD19-CD138+IL-10+ | [35] |
|  |  | Functional Bregs: CD19+CD38^hi^CD24^hi^IL-10+ | [34] |
|  |  | Bregs: CD19+CD5+CD1d+FOXP3+IL-10+ |  |
| **Monocytes** |  | Non-classical monocytes: CD14^low^CD16^hi^, IL-10+, IL-12+ or HLA-DR+ | [29,36] |
|  |  | Intermediate monocytes: CD14^hi^CD16+, IL-10+, IL-12+ or HLA-DR+ | [29,36] |
|  |  | Classical monocytes: CD14^hi^CD16-, IL-10+, IL-12+ or HLA-DR+ | [29,36] |
|  |  | M1-like monocytes: CD14^hi^CD16^hi^CD163^low^HLA-DR^hi^IL-12+ | [37] |
|  |  | M2-like monocytes: CD14^hi^CD16^low^CD163^hi^HLA-DR^low^IL-10+ | [37] |
| **Dendritic cells** |  | Tolerogenic DCs: CD11c+PD-L1+, CD11c+SLAM+, CD11c+ILT3+, CD11c+CD205+ | [30] |
|  |  | Active DCs: CD11c+HLA-DR+, CD11c+CD40+, CD11c+CD86+, CD11c+CD80+ | [30] |
| **Th1** |  | Th1-IFN-γ: CD4+Tbet+IFN-γ+ | [37-39] |
|  |  | Th1-TNF-α: CD4+Tbet+TNF-α+ | [37-39] |
| **Th2** |  | Th2-IL-13: CD4+Gata-3+IL-13+ | [37-39] |
|  |  | Th2-IL-4: CD4+Gata-3+ IL-4+ | [37-39] |
| **Th17** |  | Th-17-IL-17a: CD4+Ror-γ+IL-17a+ | [37,38,40,41] |
