# Supplementary figures and images for: Regulatory impairment in untreated Parkinson’s disease is not restricted to Tregs: other regulatory populations are also involved
Source: J Neuroinflammation. 2019 Nov 11;16:212. doi: 10.1186/s12974-019-1606-1 (PMC6849192; doi:10.1186/s12974-019-1606-1)

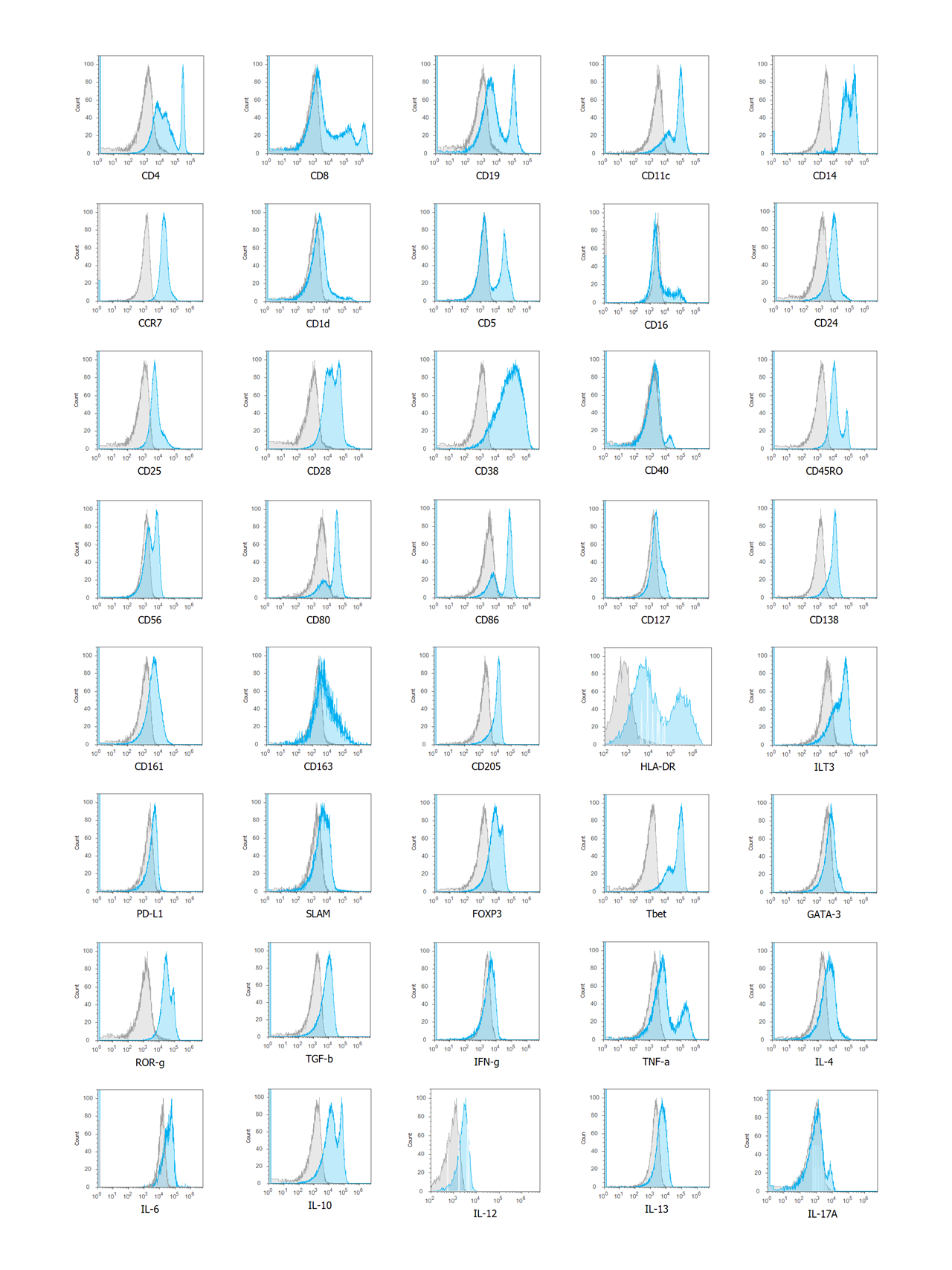

Supplement: Supplementary file 2 — Additional file 2: Figure S1. Histograms of all markers used. All antibodies used to characterize the cellular populations in our study are shown. Histograms of every marker with its respect isotype were plotted. Isotypes are shown in gray, and markers are shown in blue. [file 12974_2019_1606_MOESM2_ESM.png]
