# Supplementary material for: Regulatory impairment in untreated Parkinson’s disease is not restricted to Tregs: other regulatory populations are also involved
Source: J Neuroinflammation. 2019 Nov 11;16:212. doi: 10.1186/s12974-019-1606-1 (PMC6849192; doi:10.1186/s12974-019-1606-1)

Supplementary Figure 2. Gating strategies for Tregs, CD8regs, Bregs, monocytes, DCs, and T helper cells. **A.** CD4 regulatory T cells. The cells were characterized by a CD4 vs. size dot plot (FSC), where a region R1 was generated by selecting CD4+ cells only. A second CD25 vs. FOXP3 dot plot was generated from R1. Cells with phenotype CD4+CD25hiFOXP3+ were termed as classical Tregs. A CD45RO vs. FOXP3 dot plot was also generated from R1; CD4+CD45RO+FOXP3hi cells were termed as active Tregs, CD4+CD45RO+FOXP3med cells were called non-Tregs, and CD4+CD45RO−FOXP3low cells were named as resting Tregs. On the other hand, another FSC vs. dispersion dot plot (SSC) was generated, selecting a lymphocyte-only region labeled as R1. A CD4 vs. CD25 dot plot was generated from R1, selecting only CD4+CD25hi cells in a region named as R3. A FOXP3 vs. CD127 dot plot was generated, and CD4+CD25hiFOXP3+CD127− cells were termed as suppressive Tregs. Finally, the cells were characterized by a CD4 vs. size dot plot (FSC), where a region R1 was generated by selecting CD4+ cells only. A CD25 vs. IL-10 dot plot was also generated from R1; CD4+CD25hiIL-10+ cells were termed as Tr1 cells. A CD25 vs. TGF-β dot plot was generated from the same R1 region, and CD4+CD25hiTGF-β+ cells were named as Th3. **B.** CD8 regulatory T cells. labeling, a CD8 vs. FSC dot plot was generated, defining a region of CD8+ cells only, named as R1. A CD56 vs. CD161 dot plot was generated from R1; CD8+CD56+CD161− were regarded as cytolytic CD8regs. A CD28 vs. FOXP3 dot plot was derived from R1; CD8+CD28−FOXP3+ cells were termed as CD8regs. On the other hand, a lymphocyte region in an FSC vs. SSC dot plot was named as R1. A CD8 vs. IL-10 dot plot was generated from R1, selecting a region of CD8+IL-10+ cells only, termed as R3. A CCR7 vs. CD45RO dot plot was generated from R3; CD8+IL-10+CCR7+CD45RO+ cells were regarded as functional CD8regs. **C.** B regulatory cells. An FSC vs. SSC dot plot was generated, selecting a region named as R1. A CD19 vs. CD138 dot plot was derived from R1; CD19−CD138+ cells were selected to define the region R2. An IL-10 histogram was generated from R2; CD19−CD138+IL-10+ cells were termed as IL-10-producing plasma cells. Another phenotype was defined from a CD19 vs. FSC dot plot, a CD19+ cell region was labeled as R1. A CD38 vs. CD24 dot plot was generated from R1; CD38hiCD24hi cells were selected, generating the region R3. An IL-10 histogram was generated, and CD19+CD38hiCD24hiIL-10+ were defined as functional Bregs. A CD5 vs. CD1d dot plot was then generated from R1; CD5+CD1d+ cells defined a new region termed as R7. A FOXP3 vs. IL-10 dot plot was generated from R7; CD19+CD5+CD1d+FOXP3+IL-10+ cells were termed as Bregs.

D. Monocytes. An FSC vs. SSC dot plot was generated, and a region named as R1 was defined. A CD14 vs. CD16 dot plot was derived from R1, and three regions were defined in this plot: R2, including CD14lowCD16hi cells, also known as non-classical monocytes; R3, representing CD14hiCD16+ cells, termed as intermediate monocytes; and R4, comprising CD14hiCD16− cells, named as classical monocytes. A histogram was generated from each of these three monocyte phenotypes to determinate IL-10-, IL-12- or HLA-DR-positive cells. In a further analysis, two regions were defined from R1: R5, including CD14hiCD16hi cells, and R6, representing CD14hiCD16low cells. A CD163 vs. HLA-DR dot plot was derived from R5, defining a region of CD163lowHLA-DRhi cells, labeled as R11. An IL-12 histogram was generated from R11, obtaining CD14hiCD16hiCD163lowHLA-DRhiIL-12+ cells, termed as M1-like monocytes. Starting from R6, a CD163 vs. HLA-DR dot plot was generated and the CD163hiHLA-DRlow cells were selected, to define the region R16. An IL-10 histogram was generated from R16, obtaining CD14hiCD16lowCD163hiHLA-DRlowIL-10+ cells, named as M2-like monocytes. **E.** Dendritic cells. An FSC vs. SSC dot plot was generated, and a dendritic region was termed as R1. Several dot plots were derived from R1: 1) CD11c vs. PD-L1; 2) CD11c vs. SLAM; 3) CD11c vs. ILT3; 4) CD11c vs. CD205; 5) CD11c vs. HLA-DR; 6) CD11c vs. CD40; 7) CD11c vs. CD80; and 8) CD11c vs. CD86. CD11c+PD-L1+, CD11c+SLAM+, CD11c+ILT3+, and CD11c+CD205+ cells were labeled as tolerogenic DCs. CD11c+HLA-DR+, CD11c+CD40+, CD11c+CD80+, and CD11c+CD86+ cells were regarded as active DCs. **F.** T helper cells. Th1: To characterize these cells, a CD4 vs. FSC dot plot was generated; CD4+ cells were selected to form the region R1. Tbet vs. IFN-γ and Tbet vs. TNF-α dot plots were generated from R1 and two phenotypes were defined: CD4+Tbet+IFN-γ+ cells, named as Th1-IFN-γ cells, and CD4+Tbet+TNF-α+ cells, named as Th1-TNF-α cells. Th2 cells: To characterize these cells, a CD4 vs. FSC dot plot was generated; CD4+ cells were selected in the plot to form the region R1. GATA-3 vs. IL-13 and GATA-3 vs. IL-4 dot plots were generated from R1 to define two phenotypes: CD4+GATA-3+IL-4+ cells, termed as Th2-IL-4 cells, and CD4+GATA-3+IL-13+ cells, named as Th2-IL-13 cells. Th17 cells: To characterize these cells, a CD4 vs. FSC dot plot was generated; CD4+ cells were selected in the plot to form the region R1. ROR-γ vs. IL-17α dot plot was generated from R1, thus defining the phenotype: CD4+ROR-γ+IL-17α+ termed as Th17-IL-17α cells.

A) CD4 regulatory T cells

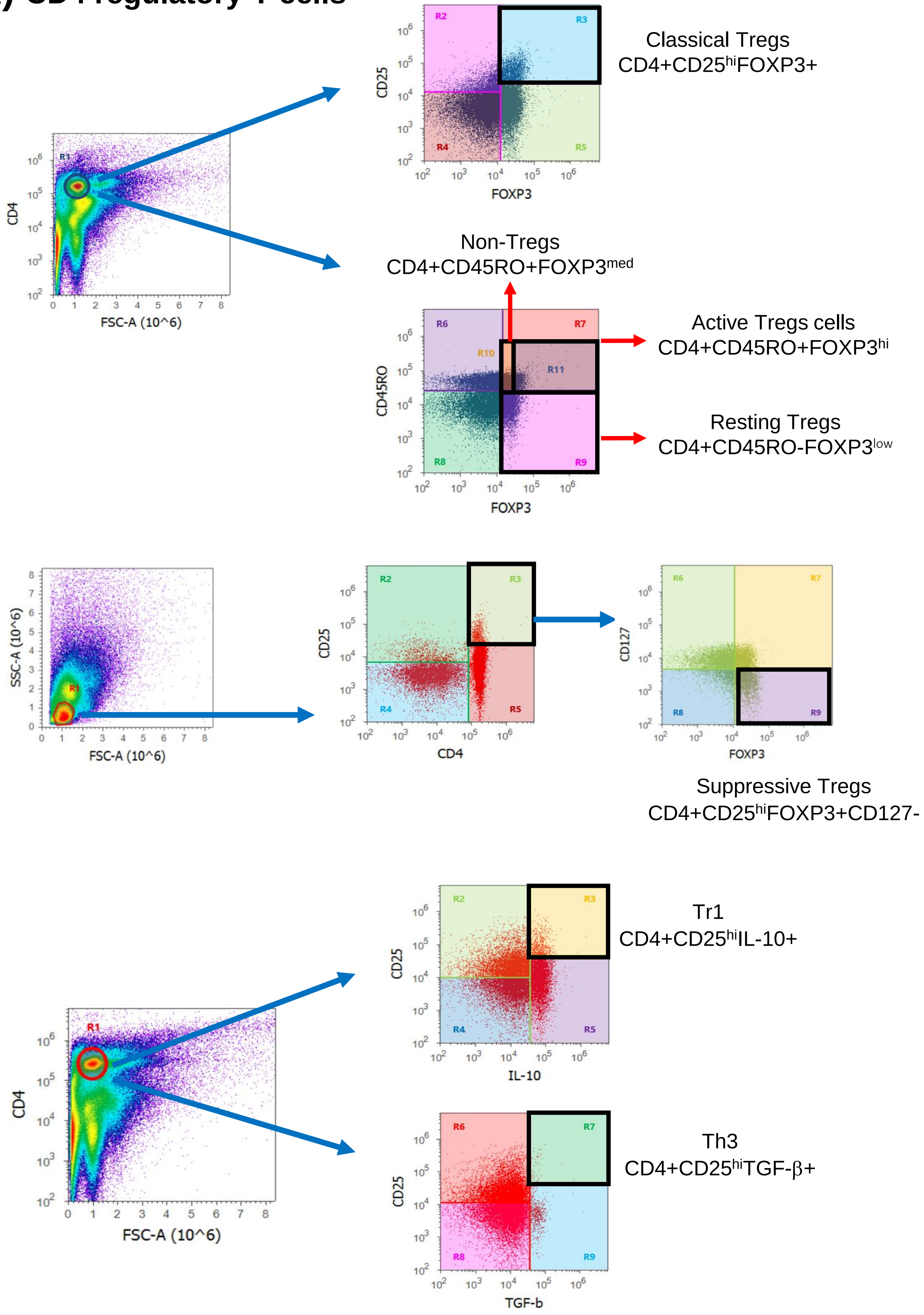

B) CD8 regulatory T cells

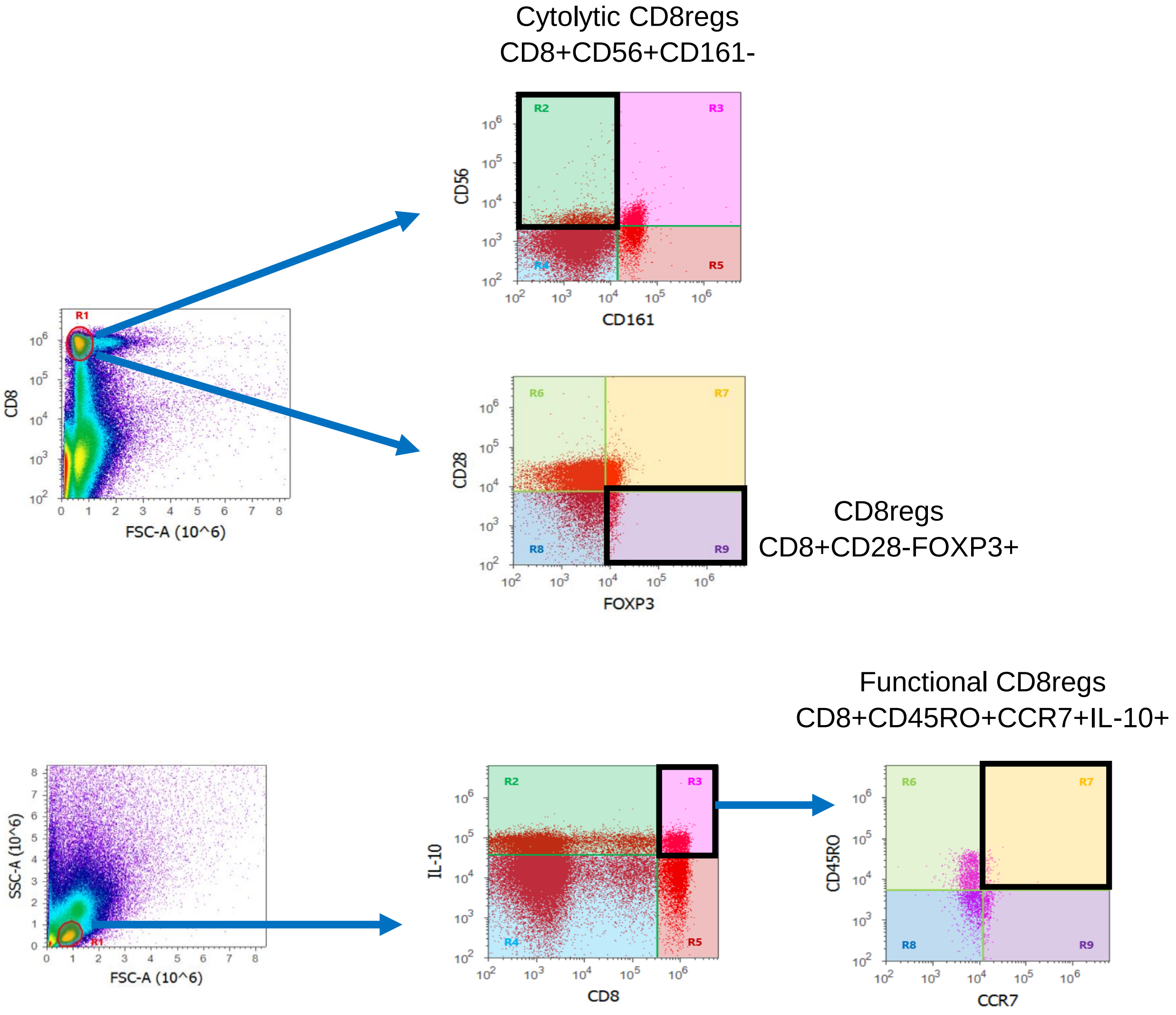

C) B regulatory cells

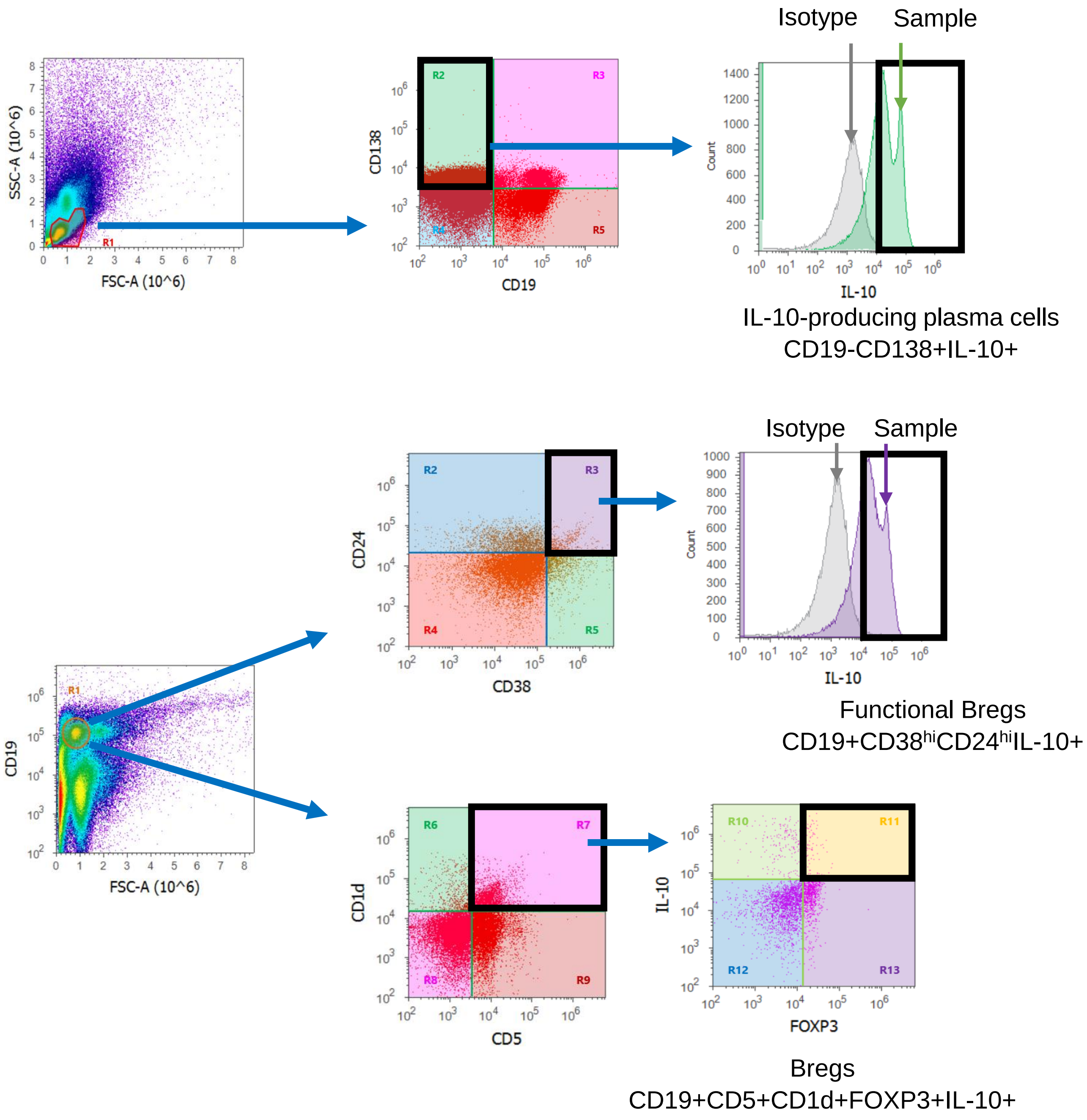

D) Monocytes

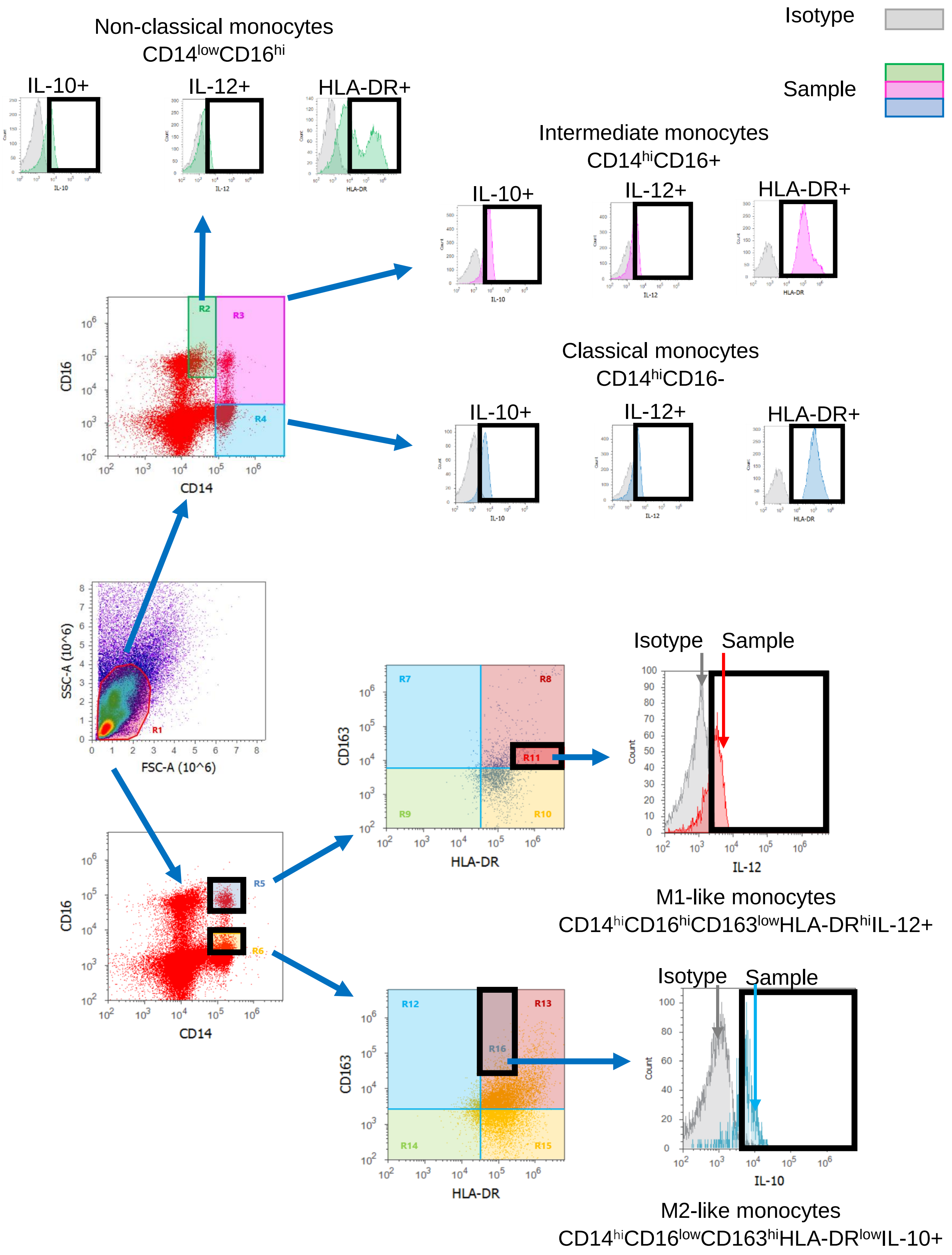

Isotype
Sample
Non-classical monocytes
CD14lowCD16hi
IL-10+
IL-12+
HLA-DR+
Intermediate monocytes
CD14hiCD16+
IL-10+
IL-12+
HLA-DR+
Classical monocytes
CD14hiCD16-
IL-10+
IL-12+
HLA-DR+
R1
R2
R3
R4
R5
R6
R7
R8
R9
R10
R11
R12
R13
R14
R15
R16
CD16
CD14
SSC-A (10^6)
FSC-A (10^6)
CD163
HLA-DR
Isotype
Sample
Count
IL-12
M1-like monocytes
CD14hiCD16hiCD163lowHLA-DRhiIL-12+
Isotype
Sample
Count
IL-10
M2-like monocytes
CD14hiCD16lowCD163hiHLA-DRlowIL-10+

E) Dendritic cells

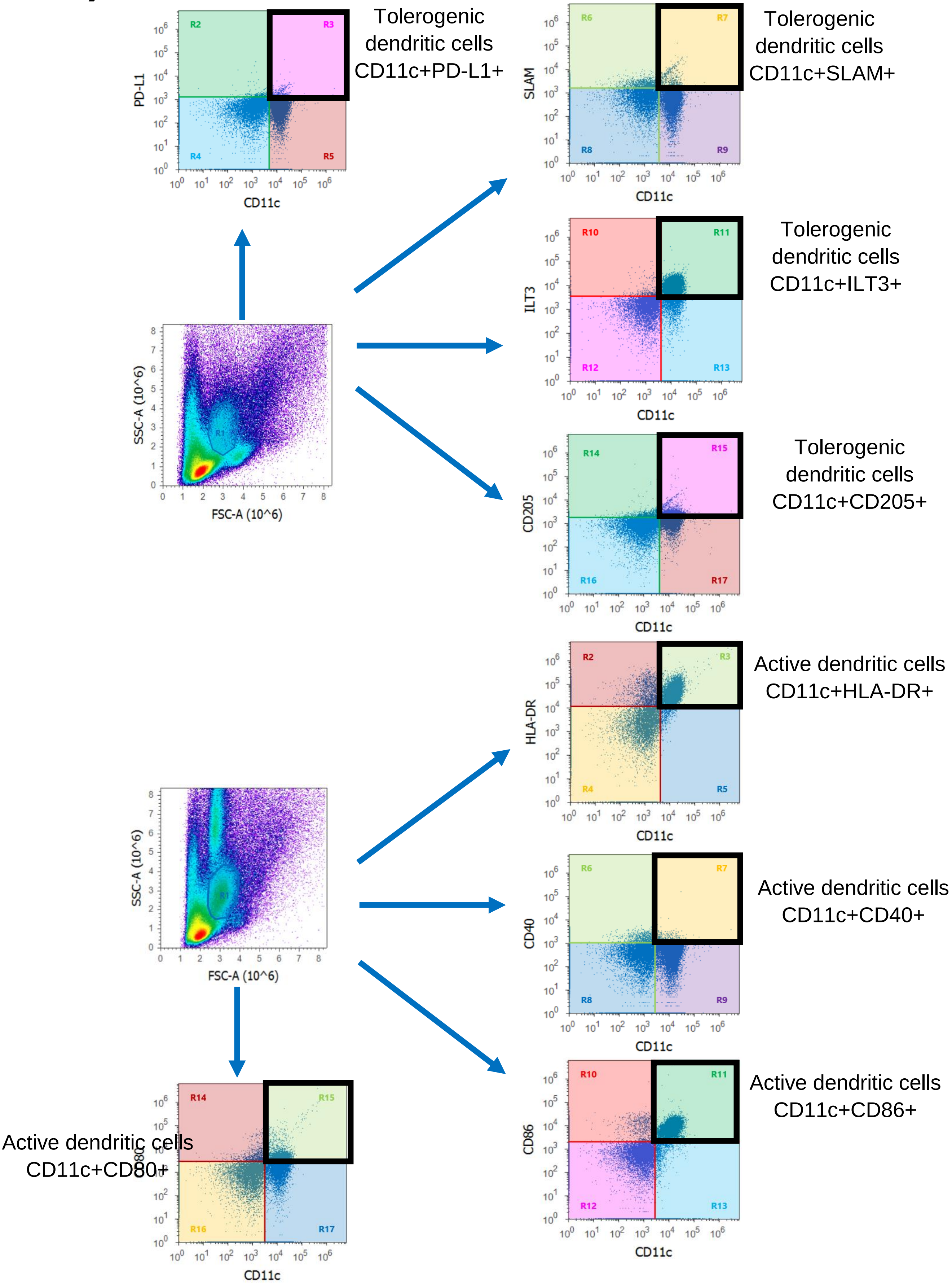

F) T helper cells

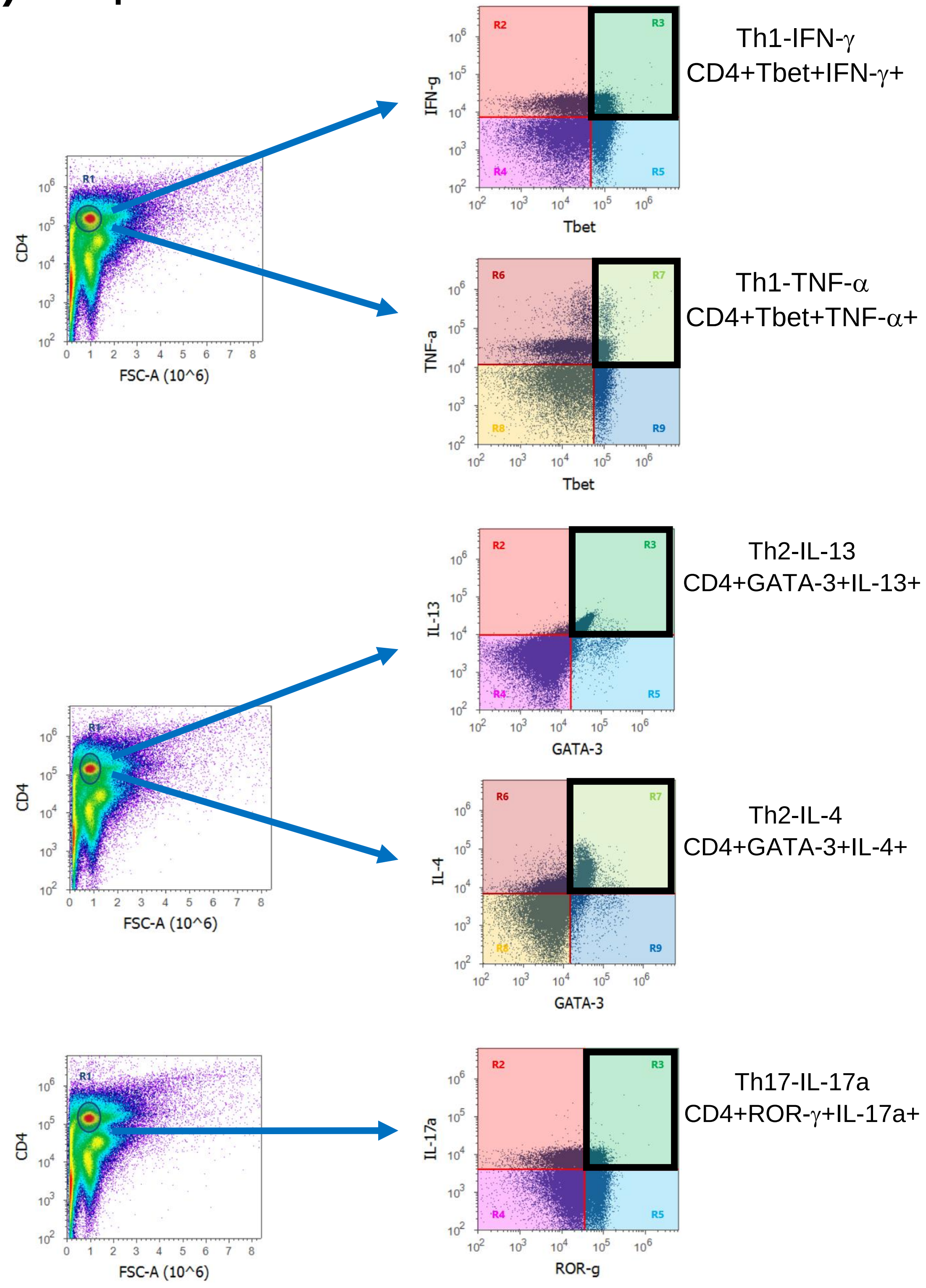

Supplement: Supplementary file 3 — Additional file 3: Figure S2. Gating strategies for Tregs, CD8regs, Bregs, monocytes, DCs, and T helper cells. A. CD4 regulatory T cells. B. CD8 regulatory T cells. C. B regulatory cells. D. Monocytes. E. Dendritic cells. F. T helper cells. [file 12974_2019_1606_MOESM3_ESM.pdf]
