# Supplementary material for: Regulatory impairment in untreated Parkinson’s disease is not restricted to Tregs: other regulatory populations are also involved
Source: J Neuroinflammation. 2019 Nov 11;16:212. doi: 10.1186/s12974-019-1606-1 (PMC6849192; doi:10.1186/s12974-019-1606-1)
Supplement: Supplementary file 4 — Additional file 4: Table S2. Biometric screening: Hemogram. Differences in the total cell blood count between patients and healthy controls are shown. [file 12974_2019_1606_MOESM4_ESM.docx]

**Supplementary Table 2. Biometric screening: Hemogram**

|  |  | **Controls^¢^** |  | **Patients^¢^** |  | ***P*** |
| --- | --- | --- | --- | --- | --- | --- |
| Total leucocytes |  | 6.05 ± 1.49 |  | 6.99 ± 6.03 |  | 0.75 |
| Absolute lymphocytes |  | 2.02 ± 0.74 |  | 1.83 ± 0.68 |  | 0.18 |
| Absolute monocytes |  | 0.41 ± 0.12 |  | 0.53 ± 0.16 |  | 0.01* |
| Absolute eosinophils |  | 0.17 ± 0.21 |  | 0.19 ± 0.19 |  | 0.50 |
| Absolute basophils |  | 0.03 ± 0.03 |  | 0.04 ± 0.03 |  | 0.65 |
| Absolute neutrophils |  | 3.42 ± 1.25 |  | 3.32 ± 1.03 |  | 0.81 |
| Total erythrocytes (U/L) |  | 5.13 ± 0.48 |  | 5.07 ± 0.45 |  | 0.44 |
| Hemoglobin (g/dL) |  | 15.37 ± 1.58 |  | 15.47 ± 1.59 |  | 0.94 |
| Hematocrit (%) |  | 45.83 ± 3.46 |  | 46.17 ± 4.33 |  | 0.96 |
| Corpuscular medium volume (CMV) (fL) |  | 89.59 ± 5.19 |  | 91.08 ± 2.94 |  | 0.21 |
| Medium concentration of corpuscular hemoglobin^^^ |  | 33.31 ± 1.55 |  | 33.49 ± 0.94 |  | 0.81 |
| Platelets (10^3^/µL) |  | 242.64 ±64.34 |  | 233.78 ± 71.8 |  | 0.25 |

^¢^Data are expressed as mean ± SD. *Values are considered as significantly different for *P* < 0.05.
