# Supplementary material for: Regulatory impairment in untreated Parkinson’s disease is not restricted to Tregs: other regulatory populations are also involved
Source: J Neuroinflammation. 2019 Nov 11;16:212. doi: 10.1186/s12974-019-1606-1 (PMC6849192; doi:10.1186/s12974-019-1606-1)
Supplement: Supplementary file 5 — Additional file 5: Table S3. Biometric screening: Blood chemistry. Differences in blood chemistry test between patients and healthy controls are shown. [file 12974_2019_1606_MOESM5_ESM.docx]

**Supplementary Table 3. Biometric screening: Blood chemistry**

|  | **Controls^¢^** | **Patients^¢^** | ***P*** |
| --- | --- | --- | --- |
| Glucose^¥^ | 98 ± 26.92 | 94.29 ± 15.66 | 0.73 |
| Blood ureic nitrogen^¥^ (BUN) | 15.94 ± 7.67 | 16.10 ± 4.40 | 0.20 |
| Urea^¥^ | 29.95 ± 8.79 | 34.55 ± 9.25 | 0.06 |
| Creatinine^¥^ | 0.89 ± 0.27 | 0.81 ± 0.17 | 0.30 |
| Uric acid^¥^ | 6.13 ± 1.5 | 5.79 ± 1.33 | 0.39 |
| Total cholesterol^¥^ | 217.35 ± 55.95 | 188.80 ± 34.67 | 0.07 |
| Triglycerides^¥^ | 162.17 ± 63.02 | 142.19 ± 56.69 | 0.30 |
| Aspartate aminotransferase^¥^ (AST) | 24.41 ± 6.50 | 23.21 ± 7.62 | 0.42 |
| Alanine aminotransferase^¥^ (ALT) | 30.53 ± 13.74 | 23.16 ± 15.79 | 0.005* |
| Total bilirubin^¥^ (BT) | 0.65 ± 0.25 | 0.56 ± 0.38 | 0.054 |
| Total protein^£^ | 7.35 ± 0.40 | 7.29 ± 0.44 | 0.36 |
| Albumin^£^ | 4.36 ± 0.44 | 4.42 ± 0.30 | 0.80 |
| Gamma-glutamyl-transpeptidase^¥^ (GGT) | 44.45 ± 41.33 | 31.50 ± 33.39 | 0.051 |
| Calcium^¥^ (Ca) | 9.32 ± 0.39 | 9.46 ±0.74 | 0.52 |
| Phosphorus^¥^ (P) | 3.43 ± 0.57 | 3.49 ± 0.52 | 0.76 |
| Sodium^¤^ (Na) | 140.01 ± 1.94 | 141.46 ± 1.77 | 0.019* |
| Potassium^¤^ (K) | 4.75 ± 0.71 | 4.30 ± 0.45 | 0.09 |
| Chlorine^¤^ (Cl) | 104.31 ± 3.06 | 101.69 ± 2.49 | 0.007* |

^¢^Data are expressed as mean ± SD. ^¥^mg/dL, ^£^g/dL, ¤Eq/dL. *Values are considered as significantly different for *P* < 0.05.
