# Supplementary material for: Regulatory impairment in untreated Parkinson’s disease is not restricted to Tregs: other regulatory populations are also involved
Source: J Neuroinflammation. 2019 Nov 11;16:212. doi: 10.1186/s12974-019-1606-1 (PMC6849192; doi:10.1186/s12974-019-1606-1)
Supplement: Supplementary file 6 — Additional file 6: Table S4. Biometric screening: Hormonal profile. Differences in the hormonal profile between patients and healthy controls are shown. [file 12974_2019_1606_MOESM6_ESM.docx]

**Supplementary Table 4. Biometric screening: Hormonal profile**

|  |  | **Controls^¢^** |  | **Patients^¢^** |  | ***P*** |
| --- | --- | --- | --- | --- | --- | --- |
| T-uptake |  | 42.17 ±1.93 |  | 42.81 ±3.74 |  | 0.49 |
| Free thyroxine |  | 11.01 ±1.66 |  | 11.46 ±1.54 |  | 0.24 |
| Free thyroxine index |  | 96.49 ±24.64 |  | 116.01 ±18.53 |  | 0.001* |
| Total thyroxine (T4) nmol/L |  | 94.91 ±22.04 |  | 109.04 ±16.87 |  | 0.012* |
| Total triiodothyronine (T3) nmol/L |  | 1.66 ± 0.30 |  | 1.57 ± 0.35 |  | 0.31 |
| Free triiodothyronine |  | 5.38 ± 1.73 |  | 4.91 ± 1.04 |  | 0.32 |
| Thyrotropin µIU/mL |  | 3.28 ± 2.61 |  | 2.92 ± 2.16 |  | 0.69 |
| Luteinizing hormone (LH) mUI/mL |  | 11.68 ± 11.91 |  | 9.79 ± 10.13 |  | 0.42 |
| Follicle stimulating hormone (FSH) mUI/mL |  | 20.96 ± 23.27 |  | 20.87 ± 24.30 |  | 0.89 |
| Estradiol pg/mL |  | 31.09 ± 32.00 |  | 17.42 ± 9.99 |  | 0.26 |
| Prolactin ng/mL |  | 8.38 ± 3.36 |  | 7.59 ± 4.96 |  | 0.34 |
| Cortisol µg/mL |  | 10.17 ± 3.79 |  | 13.34 ± 4.64 |  | 0.004* |
| Testosterone ng/mL |  | 3.30 ± 2.43 |  | 5.24 ± 1.79 |  | 0.033* |

^¢^Data are expressed as mean ± SD. *Values are considered as significantly different for *P* < 0.05.
