# Supplementary material for: Regulatory impairment in untreated Parkinson’s disease is not restricted to Tregs: other regulatory populations are also involved
Source: J Neuroinflammation. 2019 Nov 11;16:212. doi: 10.1186/s12974-019-1606-1 (PMC6849192; doi:10.1186/s12974-019-1606-1)
Supplement: Supplementary file 7 — Additional file 7: Table S5. Levels of human monocytes in PD patients and healthy controls. Differences between subpopulations of monocytes between patients and healthy controls are shown. [file 12974_2019_1606_MOESM7_ESM.docx]

**Supplementary Table 5. Levels of human monocytes in PD patients and healthy controls**

| **Cell subset** |  |  | **Monocyte** |  | **HLA-DR+** |  | **IL-12+** |  | **IL-10+** |
| --- | --- | --- | --- | --- | --- | --- | --- | --- | --- |
| Non-classic  CD14^low^CD16^hi^ | **Control^¢^** |  | 2.02 ± 1.30 |  | 1.19 ± 0.80 |  | 0.39 ± 0.39 |  | 1.54 ± 1.16 |
|  | **PD^¢^** |  | 2.65 ± 1.88 |  | 1.56 ± 1.13 |  | 0.55 ± 0.53 |  | 1.96 ± 1.37 |
|  | ***P*-value** |  | 0.292 |  | 0.287 |  | 0.266 |  | 0.287 |
|  |  |  |  |  |  |  |  |  |  |
| Intermediate  CD14^hi^CD16+ | **Control^¢^** |  | 3.36 ± 2.19 |  | 3.31 ± 2.17 |  | 0.56 ± 0.74 |  | 2.30 ± 2.00 |
|  | **PD^¢^** |  | 3.43 ± 2.01 |  | 3.38 ± 1.99 |  | 0.67 ± 0.73 |  | 2.50 ± 2.02 |
|  | ***P*-value** |  | 0.707 |  | 0.693 |  | 0.275 |  | 0.610 |
|  |  |  |  |  |  |  |  |  |  |
| Classic  CD14^hi^CD16− | **Control^¢^** |  | 3.60 ± 2.18 |  | 3.54 ± 2.17 |  | 0.10 ± 0.25 |  | 2.57 ± 2.22 |
|  | **PD^¢^** |  | 3.74 ± 3.18 |  | 3.64 ± 3.05 |  | 0.13 ± 0.26 |  | 2.95 ± 3.52 |
|  | ***P*-value** |  | 0.718 |  | 0.724 |  | 0.342 |  | 0.717 |

^¢^values are expressed as mean ± SD

**P* < 0.05 is considered as significant
